# Editorial: The varieties of contemplative experiences and practices

**DOI:** 10.3389/fpsyg.2023.1232999

**Published:** 2023-06-20

**Authors:** Sucharit Katyal, Anna-Lena Lumma, Philipp R. Goldin, Sisir Roy

**Affiliations:** ^1^Max Planck UCL Centre for Computational Psychiatry and Ageing Research, University College London, London, United Kingdom; ^2^Department of Psychology and Psychotherapy, Witten/Herdecke University, Witten, Germany; ^3^Betty Irene Moore School of Nursing, University of California Davis Medical Center, Sacramento, CA, United States; ^4^Consciousness Studies Programme, National Institute of Advanced Studies (NIAS), Bengaluru, India

**Keywords:** meditation, consciousness, mindfulness, altered state of awareness, phenomenology (of religion), contemplative practices, spiritual experiences

## Introduction

While diverse contemplative techniques are employed across a plethora of traditions around the world, contemplative research over the years has not reflected this variety. Despite exponential growth in contemplative research in recent decades, it has largely been dominated within a relatively narrow and inadequately-defined construct of contemplative practice (CP) under the umbrella term “mindfulness.” The aim of this Research Topic was to provide an avenue for understanding CP from a more diverse and inclusive perspective. We envisioned this could be done by (1) studying common systems of practice (like mindfulness) in novel settings, (2) studying a wider variety of contemplative traditions and practices, and finally, (3) drawing on psychological/phenomenological/neurobiological similarities and differences between the varieties of practices and experiences to arrive at theoretical abstractions that provide novel insight into contemplation, and more generally, human mind and consciousness. We received several articles in line with these objectives.

Two papers studied mindfulness practices in novel settings. Mortlock et al. developed the idea of “team mindfulness” training and implemented it in a high-stress military setting. They found that this approach was effective in raising individual and collective stress management skills, and contributed to an understanding of the interdependence between collective and individual mindfulness capacity development. Filipe et al. conducted a systematic review of the effects of mindfulness meditation practices on cognitive and socio-emotional skills of children 6–12 years of age and observed a “reasonably strong” effect. Future work can extend these questions to other types of CPs.

Several papers in this Research Topic reported on empirical work (qualitative, cognitive, and neuroimaging) on previously understudied contemplative practice systems. Matko et al. tested incremental effects of different components of yoga-based practices on novice participants over the course of 8 weeks. Participants were assigned to one of four conditions: mantra meditation alone, meditation plus physical yoga, meditation with yoga plus ethical education, and meditation plus ethical education. They found that wellbeing generally increased over time. However, this increase was strongest for combined interventions, particularly ones including ethical education.

Ekman et al. examined qualitative changes produced by the “Feeding Your Demons” meditation process—a secular adaptation of the traditional Tibetan Buddhist meditation practice known as Chöd or “Severance.” The findings suggest that the creative process of transforming adversity and suffering into an ally allows emotional preparation for entering objectless pristine awareness. It also leads to psychological changes such as enhanced sense of self-worth and confidence, empathy for the rejected parts of oneself, increased self-awareness, and self-compassion. On the theme of compassion, Mascaro et al. studied qualitative outcomes of Cognitively-Based Compassion Training in meditation-naive Christian healthcare chaplains. They found not only that the experience of compassion is malleable but there are a number of individual ways of practicing compassion, thus yielding novel insights about how individuals actually learn and enact compassion meditation. Understanding these processes further can provide a more refined basis for designing compassion training and integration of compassion into daily life.

Two studies undertook neurocognitive study of underexplored CPs. Examination of conscience is a CP that involves prospective (for the upcoming day) and retrospective (for the day passed) examination of daily actions. Pisapia and Dall'Avanzi investigated a 2-week digital-application-based intervention using this practice on metamemory but did not find any effects that differed from an active control group. An fMRI study investigated the effect of an 8-day Samyama meditation program—derived from the Isha Yoga system of meditation—on resting-state brain functional connectivity (Vishnubhotla et al.). They reported increased functional connectivity between the salience network and the default mode network consistent with previous work on other meditation practices (though the sample-size and demographics of their control group limit the interpretations of their findings).

Two studies investigated the Ananda Marga Tantric Yoga (AMTY) system of practices on subjective experience. Tantric Yoga practices evolved in South Asia over several millennia, developing epistemic frameworks to characterize experiential changes accompanying the practices. One such framework is that of Kundalini—an otherwise latent “energy” activatable through CPs, engendering transformative experiences. While past work has investigated Kundalini from a psychopathological perspective, Maxwell and Katyal characterize Kundalini experiences in a healthy and positive transformative contemplative setting. More generally, AMTY combines a number of different physical and mental practices that support the cultivation of a holistic goal of “self-realization.” Building on a phenomenological interpretation of Samkhya philosophy originally presented by the twentieth century philosopher and polymath Prabhat Rainjan Sarkar, Katyal develops the idea of self-realization as gradual experiential reduction of three “structures” of consciousness—the mental object, an individuality-inducing mental act, and a transpersonal/non-dual existential awareness—ultimately leading to the first-person deduction of a transcendental essence from which consciousness in its experiential form is derived. Such a framework may serve as a general basis for understanding not only how CPs provide first-person insight into consciousness but also their soteriological/spiritual objectives. A part of this framework is corroborated by Chattopadhyay who develops a Buddhist account of contemplation embedded in its broader socio-spiritual context, concluding that it culminates in transforming the experience of an (individual) “I” to a (transpersonal) “we.”

In addition to Katyal and Chattopadhyay, several other papers addressed our broader aim of expanding the scope of theoretical constructs through which to understand CPs and how they relate to consciousness. A critical lacuna we identified when initiating this Research Topic was the lack of clarity in defining the term mindfulness. Levit-Binnun et al. fill this void by proposing a “Mindfulness Map” spanning two axes: (1) specific mindfulness practices and instructions, and (2) intentions to cultivate certain experiential understandings. In their framework, intentions moderate the effect of mindfulness practices on experiential understandings. This map offers a starting point for examining the role of, among other things, contextual factors in outcomes related to mindfulness practices. Relatedly, Lundh proposes that from the lens of experimental phenomenology, contemplative practices can be regarded as an “informal” phenomenological practice derived by variations of experience and its observation. In this regard, they can be extended to more targeted development of experiential manipulation toward specific objectives. Sparby and Sacchet offer yet another broad outlook at CPs by proposing a phenomenologically-derived method for classifying meditation techniques, more generally, based on the specific set of activities and objects involved in them. Even while considering the diversity contemplative practices and systems, they suggest that meditative activities can be generalized to observing, producing and being aware—ultimately leading to a broader goal of cultivating “awareness of awareness,” namely, meta-awareness. In this regard, Meling develops the idea of awareness of awareness or “non-dual knowing” in an enactive cognition framework, with a focus on the Mahamudra tradition of Tibetan Buddhism.

## Broader implications for meditation and consciousness research

**Figure 1:**
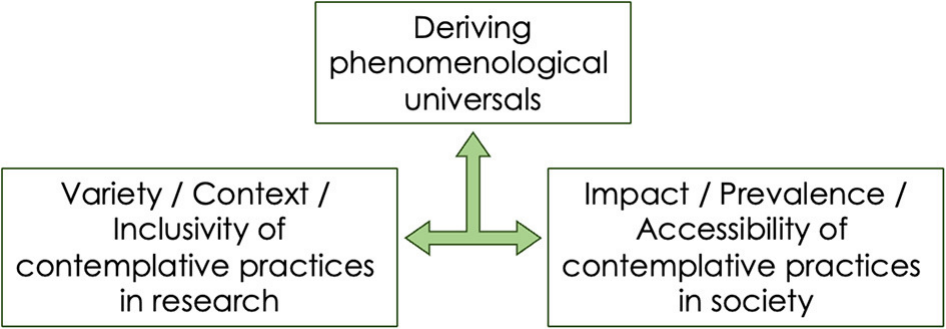
Having context, variety and inclusivity in contemplative practices will allow them to be accessible by a much wider audience. At the same time, there is better need to understand the diversity of contemplative practices prevalent already in society worldwide. Together these two can allow us to understand phenomenological universals that underlie the states attained by these practices.

One theme underlying many papers published in this Research Topic is the importance of considering the wider ethical, intentional and philosophical/spiritual context within which CPs are practiced to understand their outcomes (Chattopadhyay; Katyal; Levit-Binnun et al.; Mascaro et al.; Matko et al.; Maxwell and Katyal; Sparby and Sacchet). The importance of these contexts has gained traction in contemplative research in recent years, and these papers address this timely issue. A second theme observed across the articles is that CPs are fundamentally phenomenological exercises, and by providing systematic ways to manipulate conscious experience can offer valuable progress in our understanding of consciousness, more generally (Katyal; Levit-Binnun et al.; Lundh; Meling; Sparby and Sacchet). The second theme also relates to the folk psychological notion that most contemplative/spiritual paths lead to a common goal. As proposed by some articles in this Research Topic, this goal may pertain to a transformative experience where consciousness is experienced in a unitary- or meta-state within which all worldly experiences/dualities are constituted or dissolved. Going forward, one can envision that the field of contemplative research would need to handle the interaction of the diversity in practices, contexts and experiences on the one hand with phenomenological features that are proposed to underlie contemplative practice as universals (non-duality, existential aspects of consciousness) on the other (Figure 1). Whereas, taking context into account will enable a wider audience embedded in diverse social settings to access CPs and their benefits, the phenomenological features proposed to be inherent to the practices can ensure targeted outcomes and experiences. At the same time, the diversity and context in CPs would provide a natural tool for testing if proposed phenomenological features are indeed phenomenological universals, as articles in this Research Topic have proposed. An ambitious interpretation of the amalgamation of the diversity and unity in contemplative practices is that it could serve as a holistic framework for a global pan-contemplative and interfaith dialogue. It is however worth noting that most articles in this Research Topic were either exploratory empirical studies or theoretical in nature. Further work is needed to build on this foundation to test more planned and theoretically-grounded empirical hypotheses.

While the output of this Research Topic fulfilled some of our originally-conceived goals of understanding “*The varieties of contemplative experiences and practices*,” it is also worth mentioning that there are many contemplative systems worldwide whose understanding eludes us. A number of contemplative and mystical traditions that are widely practiced in public are acutely underrepresented in contemplative research (e.g., Sufism, Kabbalah, Taoism, and native American practices). Practically, some researchers were unable to submit articles to this Research Topic due to the high article processing charges, which may not be affordable by researchers not having research funding for open access publications—an issue that may more severely impact contemplative research due to the limited research funding available in the field.

The study of different CPs may also be relevant to broader societal issues. The use of mindfulness-based practices is well-established in areas such as health care and education. However, further research on CPs may show that certain CPs are particularly suited for specific purposes in a variety of application areas. In addition, we live in fast-paced times that will being increasingly influenced by artificial intelligence in the future, which could benefit from people becoming more aware of themselves and their relationship to the world. Future studies could for instance whether certain CPs could help people to differentiate fake content from real content and enable people to make more profound decisions. Overall, the study of CPs could also shed new light on how people can find meaning in their life despite challenges and identify sustainable solutions for individual, but also global challenges.

## Author contributions

SK contributed the summary model and figure. A-LL contributed the concluding section. All authors contributed to writing about studies in this Research Topic. All authors contributed to the article and approved the submitted version.

